# Analysis of microRNA expression in CD133 positive cancer stem‑like cells of human osteosarcoma cell line MG-63

**DOI:** 10.7717/peerj.12115

**Published:** 2021-09-03

**Authors:** Xiong Shu, Weifeng Liu, Huiqi Liu, Hui Qi, Chengai Wu, Yu-Liang Ran

**Affiliations:** 1 Beijing Research Institute of Traumatology and Orthopaedics, Beijing JiShuiTan Hospital, Beijing, China; 2 Department of Orthopaedic Oncology Surgery, Beijing Jishuitan Hospital, Peking University, Beijing, China; 3 Medical College of Qinghai University, Xining, China; 4 State Key Laboratory of Molecular Oncology, National Cancer Center/National Clinical Research Center for Cancer/Cancer Hospital, Chinese Academy of Medical Sciences and Peking Union Medical College, Beijing, China

**Keywords:** Osteosarcoma, Cancer stem cells, CD133, microRNA, miR-4284

## Abstract

Osteosarcoma (OS) is a primary malignant tumor of bone occurring in young adults. OS stem cells (OSCs) play an important role in the occurrence, growth, metastasis, drug resistance and recurrence of OS. CD133 is an integral membrane glycoprotein, which has been identified as an OSC marker. However, the mechanisms of metastasis, chemoresistance, and progression in CD133(+) OSCs need to be further explored. In this study, we aim to explore differences in miRNA levels between CD133(+) and CD133(−) cells from the MG-63 cell line. We found 20 differentially expressed miRNAs (DEmiRNAs) (16 upregulated and 4 downregulated) in CD133(+) cells compared with CD133(−) cells. Hsa-miR-4485-3p, hsa-miR-4284 and hsa-miR-3656 were the top three upregulated DEmiRNAs, while hsa-miR-487b-3p, hsa-miR-493-5p and hsa-miR-431-5p were the top three downregulated DEmiRNAs. In addition, RT-PCR analysis confirmed that the expression levels of hsa-miR-4284, hsa-miR-4485-3p and hsa-miR-3656 were significantly increased, while the expression levels of hsa-miR-487b-3p, hsa-miR-493-5p, and hsa-miR-431-5p were significantly decreased in CD133(+) cells compared with CD133(−) cells. Moreover, Kyoto Encyclopedia of Genes and Genomes (KEGG) pathway enrichment analysis revealed that predicted or validated target genes for all 20 DEmiRNAs or the selected 6 DEmiRNAs participated in the “PI3K-Akt signaling pathway,” “Wnt signaling pathway,” “Rap1 signaling pathway,” “Cell cycle” and “MAPK signaling pathway”. Among the selected six DEmiRNAs, miR-4284 was especially interesting. MiR-4284 knockdown significantly reduced the sphere forming capacity of CD133(+) OS cells. The number of invasive CD133(+) OS cells was markedly decreased after miR-4284 knockdown. In addition, miR-4284 knockdown increased the p-β-catenin levels in CD133(+) OS cells. In conclusion, RNA-seq analysis revealed DEmiRNAs between CD133(+) and CD133(−) cells. MiRNAs might play significant roles in the function of OSCs and could serve as targets for OS treatment. MiR-4284 prompted the self-renewal and invasion of OSCs. The function of miR-4284 might be associated with the Wnt signaling pathway.

## Introduction

Osteosarcoma (OS) is a primary malignant tumor of bone occurring in young adults, with a morbidity of 4,000,000 per year (Li et al., 2016; Yin et al., 2021). A combination of surgery with adjuvant and neoadjuvant chemotherapy has been developed to improve the 5-year survival rate of OS patients; nevertheless, the prognosis of OS patients remains poor due to the high rates of tumor metastasis and recurrence, and drug resistance (Liu et al., 2021; Ren et al., 2021; Yu et al., 2021; Zhang, Ma & Li, 2019a). Therefore, the molecular mechanisms underlying metastasis, chemoresistance, and progression of OS need to be clarified to improve therapeutic options.

Cancer stem cells (CSCs) are a subpopulation of tumor cells with capacities of self-renewal, differentiation, and pluripotent differentiation (Lin et al., 2021; Zhang, Ma & Li, 2019a). CSCs cause disease recurrence and chemoresistance, and lead to tumor relapse and metastasis (Camuzard et al., 2020; Tornín et al., 2021; Zhang et al., 2019b). Recently, OS stem cells (OSCs) have been shown to cause recurrence and metastasis (Wang et al., 2020a). CD133 is considered as a stem cell marker for normal and cancerous tissues (Li et al., 2013). CD133 is also identified as a CSC marker in OS (Ni et al., 2015). However, the mechanisms underlying the link between recurrence and metastasis of OS and CD133 expression in OS cells need to be further explored.

MiRNAs have been shown to regulate genes associated with the transformation, growth, apoptosis, tumorigenic ability and self-renewal capacity of OSCs (Chang et al., 2015; Yao et al., 2020; Zhang et al., 2020; Zhao et al., 2017; Zou et al., 2017). In the present study, we explore the differentially expressed miRNAs (DEmiRNAs) between CD133(+) and CD133(−) MG-63 cells, with the aim to identify possible targets for novel OS treatment strategies.

## Materials and Methods

### Cell culture

MG-63 and Saos-2 cells were provided by the China Center for Type Culture Collection as in our previous study (Shu et al., 2020). MG-63 and Saos-2 cells were cultured in RPMI‑1640 and McCoy’s 5A medium (Gibco; Thermo Fisher Scientific, Inc., Waltham, MA, USA), respectively, supplemented with 10% FBS (Gibco; Thermo Fisher Scientific, Inc., Waltham, MA, USA), 1% L‑glutamine, and 1% penicillin‑streptomycin sulfate (Thermo Fisher Scientific, Inc., Waltham, MA, USA) at 37 °C in a 5% CO_2_ humidity-controlled incubator (Chen et al., 2019; Zhou et al., 2016).

### Fluorescence‑activated cell sorting

MG-63 and Saos-2 cell suspensions were prepared by trypsinization, and 1 × 10^6^ cells in 500 μl were stained with phycoerythrin-labeled anti-CD133 (1:50, Miltenyi Biotec GmbH, Bergisch Gladbach, Germany) at 4 °C for 60 min. After washing, the CD133(+) and CD133(−) MG-63 cells were sorted by fluorescence-activated cell sorting (FACS) using a BD FACS AriaIII system (BD Biosciences).

### RNA extraction and miRNA array

Total RNA was extracted from MG-63 cells using TRIzol reagent (Takara, Japan) and the miRNeasy mini kit (Qiagen, Wset Sussex, United Kingdom) following the manufacturer’s instructions. The extracted RNAs were labeled based on the miRCURY™ Hy3™/Hy™ Power labeling kit (Exiqon, Vedbaek, Denmark) and hybridized on a miRCURY™ LNA Array (version 18.0, Exiqon, Vedbaek, Denmark). After washing, the slides were scanned using an Axon GenePix 4000B microarray scanner (Axon Instruments, Foster City, CA, USA). The scanned images were imported into the GenePix Pro 6.0 platform (Axon Instruments) for grid alignments and analysis. Replicated miRNAs were averaged, and miRNAs with expression intensities of ≥50 in all of the samples were used to calculate the normalized expression using the median normalization method. The DEmiRNAs were identified by volcano plot filtering. Finally, hierarchical clustering was performed to identify DEmiRNAs using MEV software (Version 4.6; TIGR, Microarray Software Suite 4, Boston, MA, USA).

### Prediction of target genes of DEmiRNAs and collection of validated target genes

DEmiRNA–mRNA interactions were predicted using TargetScan (http://www.targetscan.org/), miRwalk (http://www.ma.uni-heidelberg.de/apps/zmf/mirwalk/), and the miRDB (http://www.mirdb.org/) database. The validated DEmiRNA–mRNA interactions were collected from miRTarBase (https://mirtarbase.cuhk.edu.cn/).

### GO and KEGG pathway enrichment analyses of target genes of DEmiRNAs

Target genes of DEmiRNAs were obtained, and Kyoto Encyclopedia of Genes and Genomes (KEGG) pathway enrichment analysis and Gene Ontology (GO) analysis were performed using DAVID (Guo et al., 2019). The cut-off criterion for both analyses was *P* < 0.05.

### Construction of miRNA–mRNA pathway network

The association among the differently expressed miRNAs, mRNAs and the mRNA miRNAs, mRNAs and pathways in the network were represented by nodes of different shapes and colors.

### qRT-PCR

Total RNA was extracted using TRIzol reagent (Takara, Japan). Complementary DNA (cDNA) was then synthesized through reverse transcription of the RNA using the Prime-Script RT regent Kit and gDNA Eraser (TaKaRa) (Wang et al., 2020a). Subsequently, quantitative real-time PCR (qRT-PCR) was performed using the SYBR Premix Ex Taq II Kit (Takara, Kusatsu, Japan) and an ABI 7500 qRT-PCR system (Applied Biosystems, Waltham, MA, USA), using the DNA as template. *U6* was used as the internal control. The relative miRNA expression levels were calculated based on the 2^–ΔΔCt^ equation. The primers (Sangon Biotech, Shanghai, China) are shown in Table 1.

**Table 1:** The primers used in this study.

miRNA/gene	Primer
hsa-miR-431-5p	TATATGTCTTGCAGGCCGTCAT
hsa-miR-493-5p	TCGTTGTACATGGTAGGCTTTCATT
hsa-miR-487b-3p	CAATCGTACAGGGTCATCCACTT
hsa-miR-3656	TATATATATAGGCGGGTGCGGG
hsa-miR-4485-3p	TATATATAACGGCCGCGGTACC
hsa-miR-4284	TATATATAGGGCTCACATCACCCCAT

### Sphere formation assay

MG‑63 and Saos‑2 cells (500 cells/well) were plated in Ultra‑Low Attachment 24‑well plates (Corning, Inc., Corning, NY, USA) with 0.8% methyl cellulose (Sigma‑Aldrich, St. Louis, MO, USA; Merck KGaA, Darmstadt, Germany) supplemented with 20 μl/ml B27, 20 ng/ml bFGF, 10 ng/ml epidermal growth factor, 1% L‑glutamine and 1% penicillin‑streptomycin sulfate (all were obtained from Thermo Fisher Scientific, Inc., Waltham, MA, USA). Every 3 days, each well was examined under a light microscope (IX71; Olympus Corporation, Tokyo, Japan).

### Invasion assay

To assess the invasive ability, 2 × 10^5^ serum-starved cells were seeded in 200 μl medium without serum and then plated in the top of a Transwell™ chamber (24-well insert; pore size, 8 μm; Corning, Corning, NY, USA) that was coated with diluted Matrigel (BD Biosciences). After 8 h, the infiltrating cells were stained with 4,6-diamidino-2-phenylindole (DAPI) and counted using a microscope. The invasive ability of cells was quantified.

### MiR-4284 knockdown

FACS‑sorted CD133(+) and CD133(−) cells were seeded into 6-well plates and incubated overnight. They were transfected with siRNA against miR-4284 or negative control (Ribobio Co) at 60 nM using Lipofectamine 2000 (Invitrogen) according to the manufacturer’s instructions. After 72 h, the cells were harvested and RNA and protein were isolated.

### Western blot

Proteins were isolated using a Total Protein Extraction Kit (Thermo Fisher Scientific) according to the manufacturer’s protocol. The following primary antibodies were used to detect protein expression: anti-β-catenin (#8480; Cell Signaling), anti-phospho-β-catenin (#9561; Cell Signaling), and anti-β-actin (#4970; Cell Signaling). Antibodies were diluted as specified in the specifications.

### Statistical analysis

Data were analyzed using GraphPad Prism 8 (GraphPad Software, Inc., San Diego, CA, USA). Continuous data are expressed as mean ± SD. Differences between groups were analyzed using the two-tailed Student unpaired *t*-test. Results were considered statistically significant if *P* < 0.05.

## Results

### DEmiRNAs between CD133(+) and CD133(−) MG-63 cells

**Figure 1:**
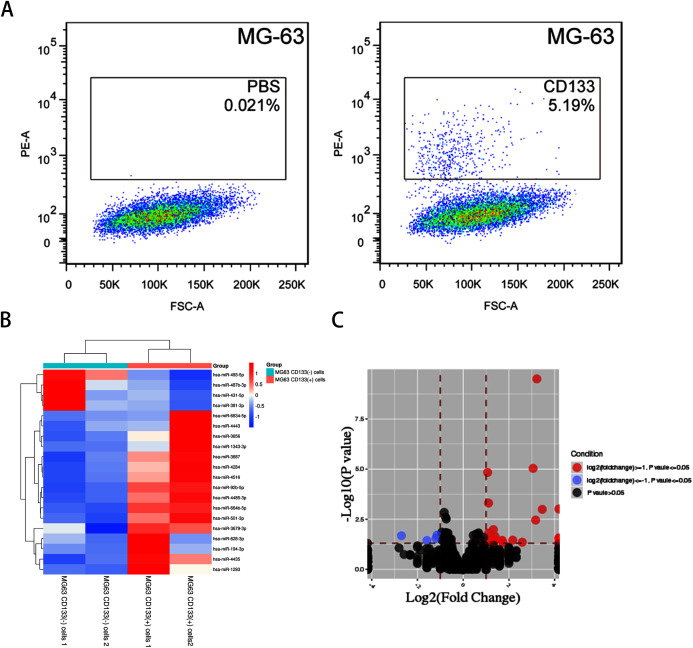
The miRNA profile in CD133(+) cells compared with CD133(–) cells from MG-63 cell line. **** (A) FACS-sorted CD133(+) cells and CD133(–) MG-63 cells. Hierarchical clustering expression (B) and volcano plots (C) showed DEmiRNAs in CD133(+) cells compared with CD133(–) cells from MG-63 cell line.

FACS-sorted CD133(+) and CD133(−) MG-63 cells (Fig. 1A) were grown under serum-free conditions for the miRNA array. DEmiRNAs between CD133(+) and CD133(−) MG-63 cells were identified. In total, 20 DEmiRNAs (fold change ≥ 1.2, *P* < 0.05) were found (16 upregulated in CD133(+) cells and 4 downregulated). Hierarchical clustering graphs and volcano plots of the 20 DEmiRNAs between CD133(+) and CD133(−) MG-63 cells are shown in Figs. 1B and 1C. The DEmiRNAs are listed in Table 2.

**Table 2:** DEmiRNAs in the MG63 CD133(+) cells compared with MG63 CD133(–) cells.

miRNA	Log (Fold)	*P* value
hsa-miR-1293	1.321928095	0.010409201
hsa-miR-1343-3p	2.169925001	0.034979785
hsa-miR-194-3p	1.378511623	0.035836906
hsa-miR-4516	3.058893689	0.003478077
hsa-miR-3679-3p	1.736965594	0.045371687
hsa-miR-3687	2.584962501	0.044350479
hsa-miR-381-3p	−1.142019005	0.01960144
hsa-miR-4284	3.459431619	0.001015678
hsa-miR-431-5p	−1.192645078	0.028247053
hsa-miR-4435	1.321928095	0.043167902
hsa-miR-4443	1.068884169	0.00001469054
hsa-miR-4485-3p	3.222392421	0.00000000031
hsa-miR-3656	3.169925001	0.00000916940
hsa-miR-487b-3p	−2.700439718	0.020999487
hsa-miR-493-5p	−1.584962501	0.036358401
hsa-miR-501-3p	1.109624491	0.000493336
hsa-miR-628-3p	1.115477217	0.015363941
hsa-miR-664b-5p	1.125530882	0.037100384
hsa-miR-6834-5p	1.700439718	0.035791281
hsa-miR-92b-5p	1.392317423	0.022664911

### Functional enrichment analysis of DEmiRNA target genes

In total, 9,687 predicted target genes of the 20 DEmiRNAs were obtained. Of these predicted target genes, experimental evidence reported in the literature was obtained for 2,099 validated target genes.

**Figure 2:**
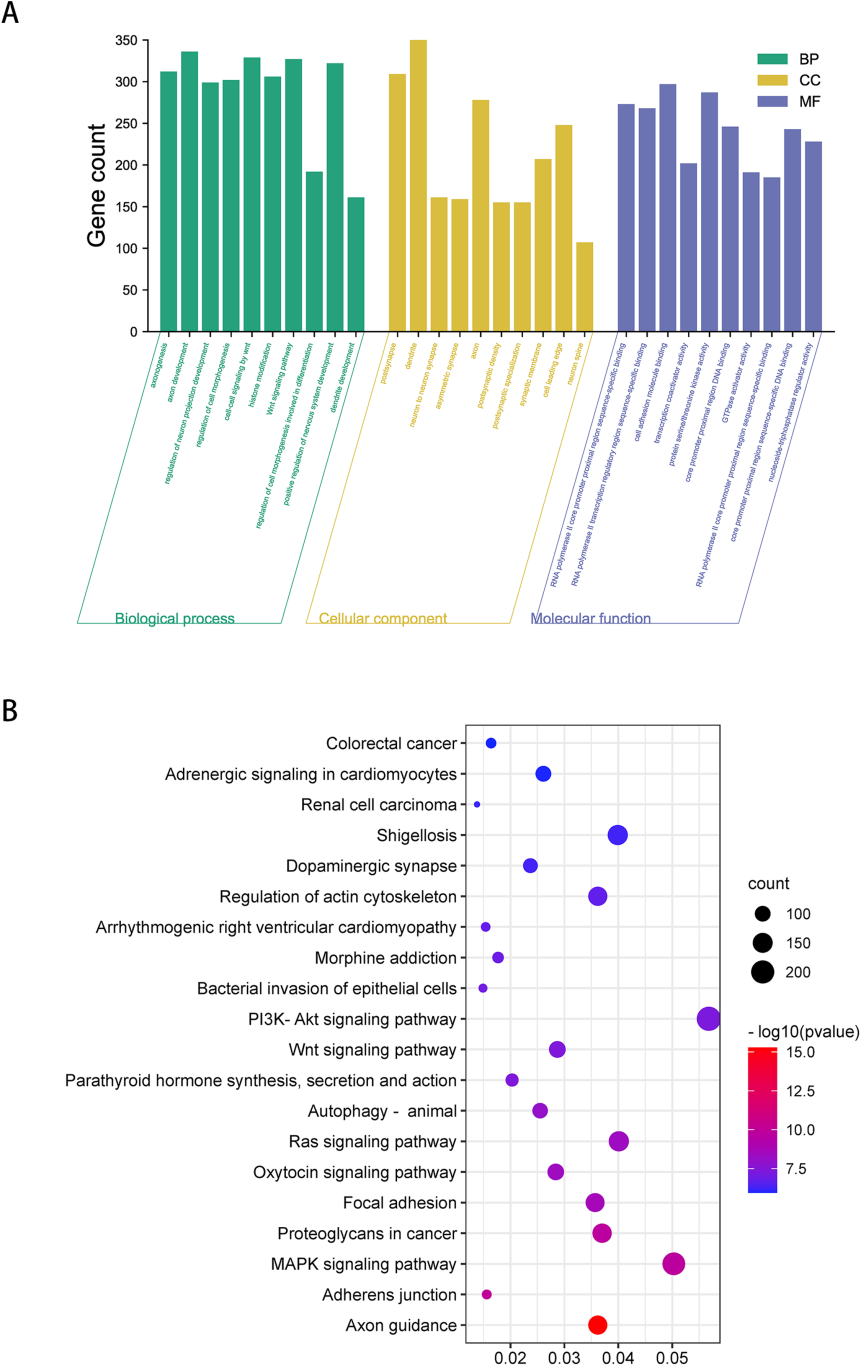
GO enrichment analysis and KEGG pathway analysis of DEmiRNAs predicted target genes. (A) Top 10 biological processes, cellular component and molecular function analysis terms of DEmiRNAs predicted target genes. (B) Top 20 KEGG pathway analysis terms of DEmiRNAs predicted target genes.

To obtain a deeper understanding of the roles of the 20 DEmiRNAs, GO enrichment analysis and KEGG pathway analysis were performed on predicted and validated target genes. GO analysis indicated that among the predicted target genes, several biological processes, including “regulation of cell morphogenesis” and “cell junction organization” (*P* < 0.05), were significantly enriched (Fig. 2A). KEGG pathway enrichment analysis indicated that among the predicted target genes, several KEGG pathways were significantly enriched (*P* < 0.05), including “Wnt signaling pathway,” “MAPK signaling pathway,” “PI3K-Akt signaling pathway,” and “Ras signaling pathway” (Fig. 2B).

**Figure 3:**
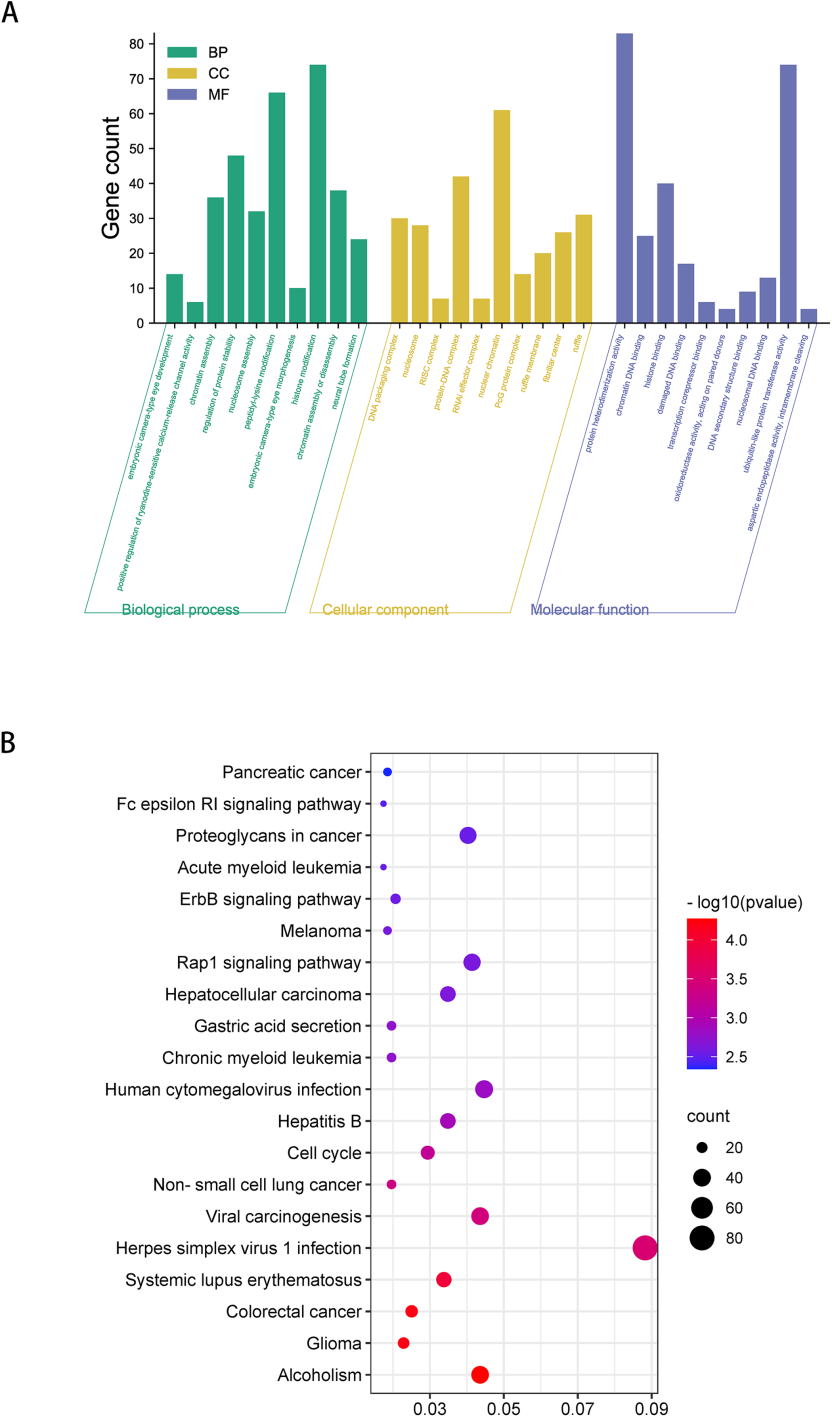
GO enrichment analysis and KEGG pathway analysis of DEmiRNAs validated target genes. (A) Top 10 biological processes, cellular component and molecular function analysis terms of DEmiRNAs validated target genes. (B) Top 20 KEGG pathway analysis terms of DEmiRNAs validated target genes.

GO analysis indicated that among the validated target genes, several biological processes, including “chromatin assembly” and “regulation of protein stability” (*P* < 0.05), were significantly enriched (Fig. 3A). KEGG pathway enrichment analysis indicated that among the validated target genes, several pathways were statistically enriched (*P* < 0.05), including “Cell cycle,” “Fc epsilon RI signaling pathway,” “ErbB signaling pathway,” and “Rap1 signaling pathway” (Fig. 3B).

### Validation and functional exploration of the top three upregulated miRNAs and top 3 downregulated miRNAs

**Figure 4:**
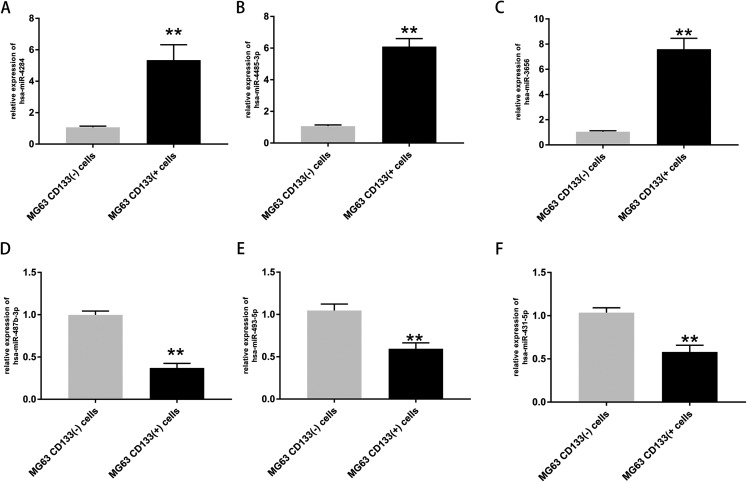
Expression of top three upregulated miRNAs and top three downregulated miRNAs. Expression of hsa-miR-4284 (A), hsa-miR-4485-3p (B), hsa-miR-3656 (C), hsa-miR-487b-3p (D), hsa-miR-493-5p (E) and hsa-miR-431-5p (F) in the MG63 CD133(+) cells compared with MG63 CD133(–) cells. **p < 0.01.

The expression of the top three upregulated miRNAs and top three downregulated miRNAs was further validated *via* RT-PCR. The results confirmed that the expression levels of hsa-miR-4284, hsa-miR-4485-3p, and hsa-miR-3656 were significantly increased in CD133(+) cells compared with CD133(−) cells (Figs. 4A–4C), while the expression levels of hsa-miR-487b-3p, hsa-miR-493-5p, and hsa-miR-431-5p were significantly decreased in CD133(+) cells compared with CD133(−) cells (Figs. 4B–4F).

**Figure 5:**
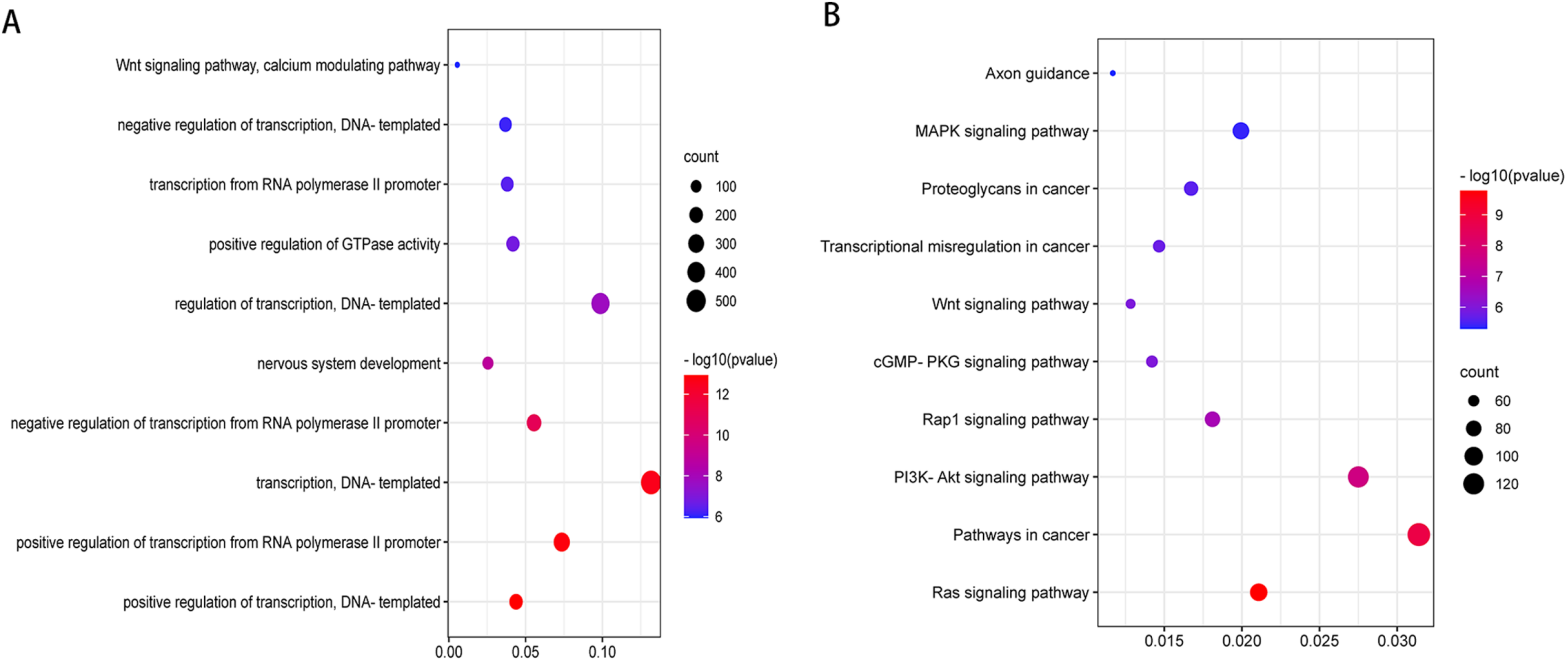
Top 10 biological processes (A) and KEGG pathway (B) analysis terms of top three upregulated miRNAs and top three downregulated miRNAs predicted target genes.

**Figure 6:**
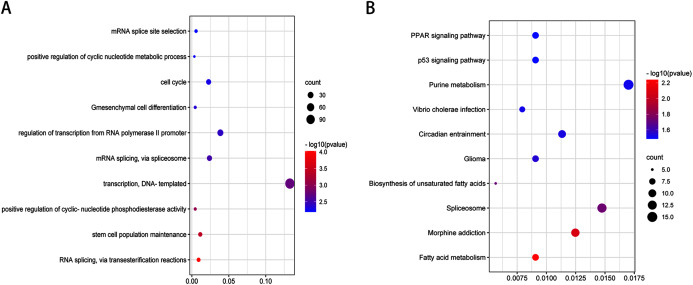
Top 10 biological processes (A) and KEGG pathway (B) analysis terms of top three upregulated miRNAs and top three downregulated miRNAs validated target genes.

After validation by RT-PCR, GO analysis and KEGG pathway enrichment analysis of the predicted and validated target genes of the top three upregulated miRNAs and top three downregulated miRNAs were also performed (Figs. 5 and 6). GO analysis indicated that among the predicted target genes, several biological, including biological processes such as “Wnt signaling pathway,” “calcium modulating pathway,” “transcription from RNA polymerase II promoter,” and “positive regulation of GTPase activity” (*P* < 0.05), were significantly enriched (Fig. 5A). KEGG pathway enrichment analysis indicated that among the predicted target genes, several KEGG pathways were significantly enriched (*P* < 0.05), including “Wnt signaling pathway,” “MAPK signaling pathway,” “Rap1 signaling pathway,” “PI3K-Akt signaling pathway,” “cGMP-PKG signaling pathway,” and “Ras signaling pathway” (Fig. 5B). GO analysis indicated that among the validated target genes, several biological processes, including “cell cycle,” “stem cell population maintenance,” and “mesenchymal cell differentiation” (*P* < 0.05), were significantly enriched (Fig. 6A). KEGG pathway enrichment analysis indicated that among the validated target genes, several pathways were statistically enriched (*P* < 0.05), including “p53 signaling pathway” and “PPAR signaling pathway” (Fig. 6B). The results of the functional enrichment analysis of these six confirmed that DEmiRNA target genes are in agreement with the functional enrichment analysis of the total number of DEmiRNA target genes.

We observed a significant interconnection between the molecular roles and signaling pathways of predicted and validated target mRNAs (Tables S1 and S2). For instance, in the interconnection among predicted target mRNAs and multiple signaling pathways, WNT7A and FZD5 regulates “Wnt signaling pathway,” “Proteoglycans in cancer,” and “Pathways in cancer”. IGF1R regulates “Proteoglycans in cancer,” “PI3K-Akt signaling pathway,” “Pathways in cancer,” “Ras signaling pathway,” “Rap1 signaling pathway,” and “Transcriptional misregulation in cancer”. AKT3 regulates “PI3K-Akt signaling pathway,” “Ras signaling pathway,” “Pathways in cancer,” “Rap1 signaling pathway,” “cGMP-PKG signaling pathway,” “Proteoglycans in cancer,” and “MAPK signaling pathway”. IGF1R is the predicted target gene for miR-493-5p and miR-431-5p. AKT3 is the predicted target gene of hsa-miR-493-5p. In the interconnection among validated target mRNAs and multiple signaling pathways, CDKN1A and CDK4 regulate the p53 signaling pathway. WNT7A and FZD5 are predicted target genes of hsa-miR-4284. CDKN1A is a validated target gene for hsa-miR-493-5p. CDK4 is a validated target gene for hsa-miR-431-5p and hsa-miR-4284.

### MiR-4284 regulates the self-renewal capacity and invasion ability in CD133(+) cells through Wnt signaling

**Figure 7:**
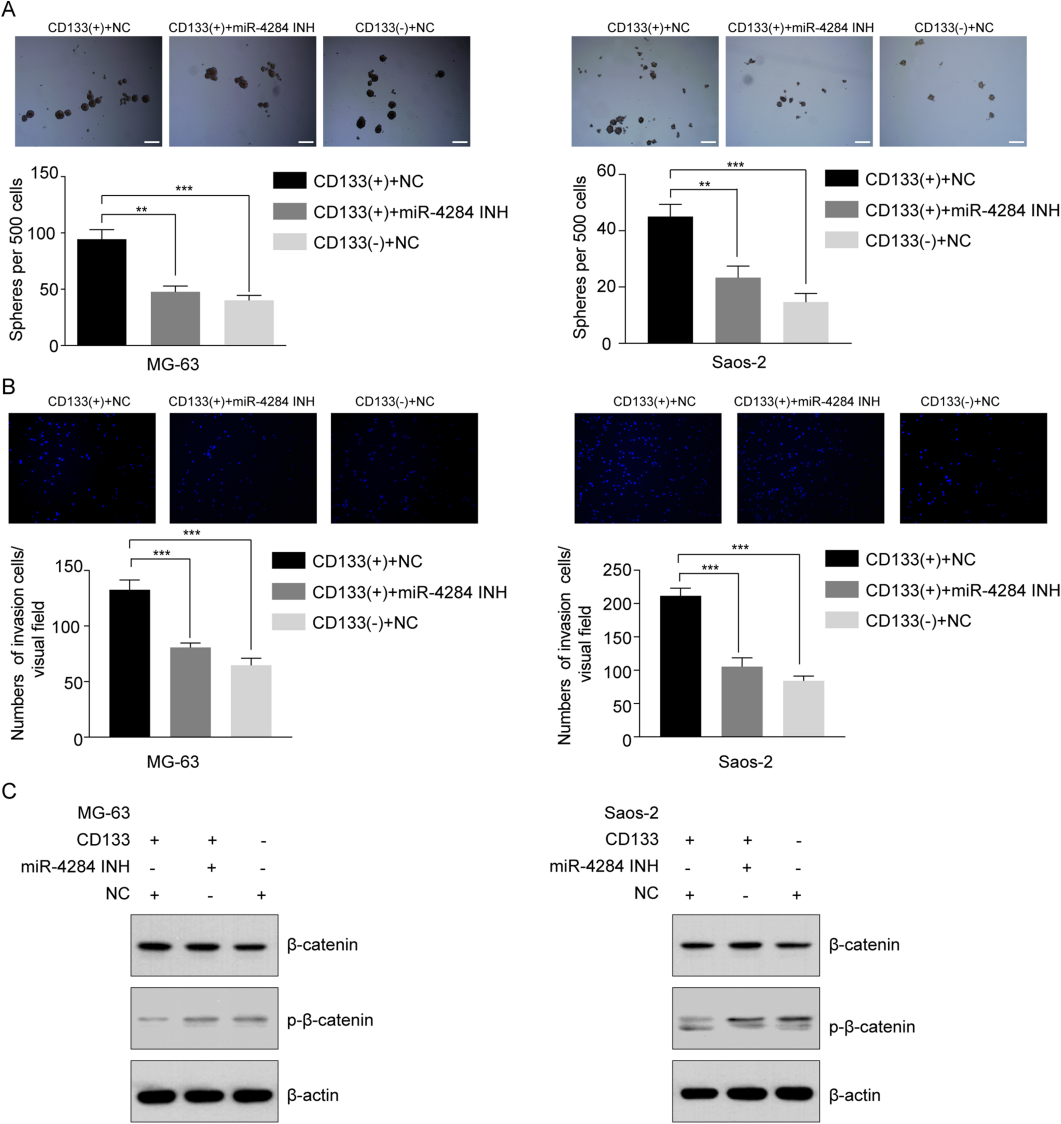
Relationship between miR-4284 and the self-renewal and invasion ability and Wnt pathway in CD133(+) cells from MG-63 and Saos-2 cells. (A) Sphere formation in CD133(+), CD133(–) cells transfected with negative control and CD133(+) cells transfected with miR-4284 inhibitor in MG-63 and Saos-2 cells were identified in a low attachment plate with serum-free media. (B) The invasion ability in CD133(+), CD133(–) cells transfected with negative control and CD133(+) cells transfected with miR-4284 inhibitor in MG-63 and Saos-2 cells were identified using Matrigel-coated invasion assay. (C) Western blot analyses of CD133(+), CD133(–) cells transfected with negative control and CD133(+) cells transfected with miR-4284 inhibitor for the Wnt pathway. **p < 0.01, ***p < 0.001.

To evaluate the effect of miR-4284 on the cells’ self-renewal ability, we initially performed a sphere formation assay of CD133(+) and CD133(−) cells transfected with siRNA targeting miR-4284. MiR-4284 knockdown significantly reduced the sphere forming capacity of CD133(+) from MG-63 and Saos-2 cells (Fig. 7A). Next, we investigated the role of miR-4284 in the regulation of the invasion ability of OSCs. After 48 h, the number of invading cells in each group was calculated. The results showed that the number of invasive CD133(+) MG-63 and Saos-2 cells was markedly decreased after miR-4284 knockdown (Fig. 7B).

Next, we investigated the relationship between miR-4284 and β-catenin phosphorylation. As shown in Fig. 7C, the level of phospho-β-catenin was upregulated, while the expression level of total β-catenin remained unaltered. Collectively, these data demonstrate that miR-4284 may promotes the self-renewal ability and invasion of OCSCs through Wnt signaling.

## Discussion

OS, the most common primary bone tumor, principally arises in the long bones of children and young adults. However, the molecular mechanisms underlying the development of OS are not well clarified. Revealing these processes may uncover novel targets for the prevention and treatment of OS.

MiRNAs have been shown to regulate genes associated with tumour growth and progression of OS (Dai et al., 2021; Gong, Wei & Liu, 2021; Wang et al., 2020b). For example, decreased miR-1274a levels have been associated with tumor suppression in OS (Feng et al., 2021). Upregulation of miR-128-3p improved resistance to cisplatin in 143B and MG-63 cell lines and miR-128-3p might function as an oncogene in OS (Zhu et al., 2021). CSCs are considered as the main cause of metastasis and recurrence (Wang et al., 2020a). Moreover, miRNAs have been shown to be involved in the transformation, growth, apoptosis, invasion, self-renewal capacity and tumorigenic ability of OSCs (Chang et al., 2015; Yao et al., 2020; Zhang et al., 2020; Zhao et al., 2017; Zou et al., 2017). In OSCs, the TGFβ–miR-499a–SHKBP1 axis orchestrates the epithelial-mesenchymal transition (EMT)–associated kinase switch which contributes to resistance to EGFR inhibitors (Wang et al., 2019). Altered miR-19a levels play key roles in OSCs, partly *via* targeting the phosphatase and tensin homolog deleted on chromosome 10(PTEN) (Zhao et al., 2017). MiR-34a may inhibit OS metastasis and growth by decreasing the cells’ self-renewal and invasive capacities, as it eliminates the tumorigenic ability of OS *in vitro* (Zou et al., 2017). These studies suggest the important roles of miRNAs in OSCs and OS.

CD133, CD26, ALDH1, CD117 and Stro-1 have been reported as the OSC markers (Adhikari et al., 2010; Bao, Cheng & Chai, 2018; Czarnecka et al., 2020; Ni et al., 2015; Saini et al., 2012). CD133(+) OS cells exhibit stem-like gene expression, act as tumorinitiating cells, and play a role in the development of drug resistance and metastasis (Czarnecka et al., 2020). Lower CD24 and CD44 expression was observed in spheres as compared with monolayers in CHA59, Saos-2 and HuO9 OS cells and a significant decrease in CD24 and CD44 expression accompanied sphere culture (Saini et al., 2012). Yao et al. (2020) found a positive correlation between high expression levels of miR-155 and upregulation of CSC surface markers (CD133 and CD24) in U2OS cells. Aldehyde dehydrogenase 1 (ALDH1) is another commonly used biomarker of CSCs in a variety of human cancers, including OS (Bao, Cheng & Chai, 2018; Ni et al., 2015). The activation of ALDH1+CD133+ cells in OS is accompanied by the downregulation of miR-143 expression (Zhou et al., 2015). CD117 and Stro-1 can be used to identify OSCs associated with the most lethal characteristics of the disease—metastasis and drug resistance—and these markers are promising candidate targets for OSC-targeted drug delivery (Adhikari et al., 2010). MiRNAs are differentially expressed CD117+stro-1+ and CD117−stro-1− OS cells, and miR-15a, miR-302a, miR-423-5p, miR-212, miR-1247, miR-518b, miR-890, and miR-1243 are DEmiRNAs (Zhao et al., 2015). The exact roles of these markers in OSCs still need to be further studied.

In the present study, we uncovered 20 DEmiRNAs between CD133(+) and CD133(−) MG-63 cells (16 upregulated in CD133(+) cells and 4 downregulated). Hsa-miR-4485-3p, hsa-miR-4284, and hsa-miR-3656 were the top three upregulated DEmiRNAs, while hsa-miR-487b-3p, hsa-miR-493-5p and hsa-miR-431-5p were the top three downregulated DEmiRNAs. The significantly differential expression of these six DEmiRNAs was confirmed by RT-PCR analysis. In a previous study, upregulation of miR-487b-3p inhibited OS formation and the combination treatment of doxorubicin and miR-487b-3p significantly inhibited CSC-induced tumor growth (Cheng et al., 2020). The level of circulating miR-493-5p is a novel potential diagnostic biomarker for OS (Huang et al., 2019). Overexpression of miR-493-5p inhibits metastasis and proliferation of OS cells by inactivating the PI3K/AKT signaling pathway and downregulating KLF5 (Zhang et al., 2019b). Upregulation of miR-431-5p inhibits OS the tumorigenesis *via* targeting PANX3 (Sun et al., 2020). The roles of these six DEmiRNAs in OS and OSCs still require further exploration.

Furthermore, we found that predicted target genes of the 20 identified DEmiRNAs were enriched in several biological processes, including “regulation of cell morphogenesis” and “cell junction organization.” KEGG analysis of the predicted target genes of the 20 identified DEmiRNAs revealed several significantly enriched pathways, including the “Wnt signaling pathway,” “Ras signaling pathway,” “PI3K-Akt signaling pathway,” and “MAPK signaling pathway.” Among the validated target genes, several biological processes, including “regulation of protein stability” and “chromatin assembly,” were significantly enriched. KEGG pathway enrichment analysis of the validated target genes revealed several significantly enriched pathways, including “Cell cycle,” “Rap1 signaling pathway,” “Fc epsilon RI signaling pathway,” and “ErbB signaling pathway.”

Of the top three upregulated miRNAs and top three downregulated miRNAs, miR-4284 was particularly interesting. A recent study found that miR-4284 could promote gastric tumor cell growth, migration and invasion by directly targeting TET1 (Li et al., 2018). In addition, miR-4284 was shown to promote the development of diffuse large B-cell lymphoma (Tamaddon et al., 2016). The expression of miR-4284 was also increased in clinical samples and cell lines of non-small cell lung cancer (NSCLC), and knockdown of miR-4284 inhibited the proliferation, migration and invasion of tumor cells (Tian, Wang & Du, 2021). These studies suggest that miR-4284 promotes the development of gastric cancer, diffuse large B-cell lymphoma, and NSCLC. In the present study, we found that miR-4284 expression was higher in CD133(+) than in CD133(−) cells from MG-63 cells. We further found that miR-4284 knockdown significantly reduced the sphere forming capacity of CD133(+) OS cells and the number of invasive CD133(+) OS cells markedly decreased after miR-4284 knockdown. Our present results suggest that miR-4284 prompts the self-renewal and increases the invasive ability of OSCs.

The Wnt signaling pathway plays important roles in the progression and development of many cancers including OS (Liang et al., 2021; Taciak et al., 2018). The Wnt signaling pathway has been reported to be activated in OS (Chen et al., 2015). LncRNA MRPL23-AS1 promotes tumor progression and carcinogenesis in OS by activating Wnt/β-catenin signaling *via* inhibiting miR-30b and upregulating MYH9 (Zhang et al., 2021). Blocking Wnt/LRP5 signaling modulates the EMT and suppresses the activity of metalloproteinases in OS Saos-2 cells (Guo et al., 2007). It has also been reported that Wnt signaling is only activated in the CSC subpopulation of OS cells (Singla et al., 2020). In the present study, the Wnt signaling pathway was significantly enriched among the predicted target genes of the 20 identified DEmiRNAs. β-Catenin is a significant downstream effector of the Wnt signaling pathway (Pinczewski et al., 2021). In a previous study, the endogenous p-LRP6 level was significantly increased after subarachnoid hemorrhage and further augmented after the administration of HLY78, which resulted in activation of the Wnt pathway by inhibition of the phosphorylation of β-catenin, Bax, and cleaved caspase 3 (Luo et al., 2020). Inhibiting β-catenin phosphorylation in glioma stem cells can lead to activation of the Wnt signaling pathway (Zhang et al., 2018). In the present study, miR-4284 knockdown increased the expression of p-β-catenin in CD133(+) OS cells, suggesting that miR-4284 may inhibit the Wnt signaling pathway by promoting β-catenin phosphorylation in OSCs.

In the present study, the identification of DEmiRNAs between CD133(+) and CD133(−) cells from MG-63 cells provided valuable insight regarding the stemness of OS cells. Nowadays, the number of identified miRNAs is growing quickly and hence further studies will be needed to explore their molecular and biological functions in OS. However, there are several limitations to this study. First, we studied the miRNA profile of OSCs. The expression of selected miRNAs in patient samples remains to be studied. Second, the roles of miR-4284 in CD133(+) cells were preliminarily explored, and the functional mechanism of miR-4284 needs to be further studied.

In conclusion, DEmiRNAs between CD133(+) and CD133(−) MG-63 cells were identified. MiRNAs might play significant roles in the function of OSCs and serve as potential targets for OS treatment. Moreover, miR-4284 promoted the self-renewal and invasion of OSCs. The function of miR-4284 might be associated with the Wnt signaling pathway.

## Supplemental Information

10.7717/peerj.12115/supp-1 Supplemental Information 1 Raw data.

10.7717/peerj.12115/supp-2 Supplemental Information 2 Uncropped Gel Blots.

10.7717/peerj.12115/supp-3 Supplemental Information 3 Representative 10 KEGG pathway terms with significantly enriched function for predicted targets of top 3 upregulated miRNAs and top 3 downregulated miRNAs.

10.7717/peerj.12115/supp-4 Supplemental Information 4 Representative 10 KEGG pathway terms with significantly enriched function for validated targets of top 3 upregulated miRNAs and top 3 downregulated miRNAs.

## Additional Information and Declarations

### Data Availability

The following information was supplied regarding data availability: The raw measurements are available in the Supplementary Files.

## References

[ref-1] Adhikari AS, Agarwal N, Wood BM, Porretta C, Ruiz B, Pochampally RR, Iwakuma T (2010). CD117 and Stro-1 identify osteosarcoma tumor-initiating cells associated with metastasis and drug resistance. Cancer Research.

[ref-2] Bao Z, Cheng Z, Chai D (2018). The expressions of CD133, ALDH1, and vasculogenic mimicry in osteosarcoma and their clinical significance. International Journal of Clinical and Experimental Pathology.

[ref-3] Camuzard O, Trojani MC, Santucci-Darmanin S, Pagnotta S, Breuil V, Carle GF, Pierrefite-Carle V (2020). Autophagy in osteosarcoma cancer stem cells is critical process which can be targeted by the antipsychotic drug thioridazine. Cancers (Basel).

[ref-4] Chang Y, Zhao Y, Gu W, Cao Y, Wang S, Pang J, Shi Y (2015). Bufalin inhibits the differentiation and proliferation of cancer stem cells derived from primary osteosarcoma cells through Mir-148a. Cellular Physiology and Biochemistry.

[ref-5] Chen R, Huang LH, Gao YY, Yang JZ, Wang Y (2019). Identification of differentially expressed genes in MG63 osteosarcoma cells with drug‐resistance by microarray analysis. Molecular Medicine Reports.

[ref-6] Chen C, Zhao M, Tian A, Zhang X, Yao Z, Ma X (2015). Aberrant activation of Wnt/β-catenin signaling drives proliferation of bone sarcoma cells. Oncotarget.

[ref-7] Cheng M, Duan PG, Gao ZZ, Dai M (2020). MicroRNA‐487b‐3p inhibits osteosarcoma chemoresistance and metastasis by targeting ALDH1A3. Oncology Reports.

[ref-8] Czarnecka AM, Synoradzki K, Firlej W, Bartnik E, Sobczuk P, Fiedorowicz M, Grieb P, Rutkowski P (2020). Molecular biology of osteosarcoma. Cancers (Basel).

[ref-9] Dai J, Lu L, Kang L, Zhang J (2021). MiR-501-3p promotes osteosarcoma cell proliferation, migration and invasion by targeting BCL7A. Human Cell.

[ref-48] Feng XT, Wang C, Zhang FJ, Wu XQ, Zhang Z (2021). MicroRNA-1274a serves as a prognostic biomarker in patients with osteosarcoma and is involved in tumor progression via targeting ADAM9. Journal of Biological Regulators and Homeostatic Agents.

[ref-10] Gong Y, Wei Z, Liu J (2021). MiRNA-1225 inhibits osteosarcoma tumor growth and progression by targeting YWHAZ. OncoTargets and Therapy.

[ref-11] Guo A, Wang W, Shi H, Wang J, Liu T (2019). Identification of hub genes and pathways in a rat model of renal ischemia-reperfusion injury using bioinformatics analysis of the gene expression omnibus (GEO) dataset and integration of gene expression profiles. Medical Science Monitor.

[ref-12] Guo Y, Zi X, Koontz Z, Kim A, Xie J, Gorlick R, Holcombe RF, Hoang BH (2007). Blocking Wnt/LRP5 signaling by a soluble receptor modulates the epithelial to mesenchymal transition and suppresses met and metalloproteinases in osteosarcoma Saos-2 cells. Journal of Orthopaedic Research.

[ref-13] Huang C, Wang Q, Ma S, Sun Y, Vadamootoo AS, Jin C (2019). A four serum-miRNA panel serves as a potential diagnostic biomarker of osteosarcoma. International Journal of Clinical Oncology.

[ref-49] Li J, Zhong XY, Li ZY, Cai JF, Zou L, Li JM, Yang T, Liu W (2013). CD133 expression in osteosarcoma and derivation of CD133^+^ cells. Molecular Medicine Reports.

[ref-14] Li K, Li X, Tian J, Wang H, Pan J, Li J (2016). Downregulation of DNA-PKcs suppresses P-gp expression via inhibition of the Akt/NF-κB pathway in CD133-positive osteosarcoma MG-63 cells. Oncology Reports.

[ref-15] Li Y, Shen Z, Jiang H, Lai Z, Wang Z, Jiang K, Ye Y, Wang S (2018). MicroRNA‐4284 promotes gastric cancer tumorigenicity by targeting ten-eleven translocation 1. Molecular Medicine Reports.

[ref-16] Liang K, Liao L, Liu Q, Ouyang Q, Jia L, Wu G (2021). microRNA-377-3p inhibits osteosarcoma progression by targeting CUL1 and regulating Wnt/β-catenin signaling pathway. Clinical & Translational Oncology.

[ref-17] Lin J, Wang X, Wang X, Wang S, Shen R, Yang Y, Xu J, Lin J (2021). Hypoxia increases the expression of stem cell markers in human osteosarcoma cells. Oncology Letters.

[ref-18] Liu H, Yang M, Zhang Y, Yang Z, Chen Z, Xie Y, Peng B, Cai L (2021). The effect of miR-539 regulating TRIAP1 on the apoptosis, proliferation, migration and invasion of osteosarcoma cells. Cancer Cell International.

[ref-19] Luo X, Li L, Xu W, Cheng Y, Xie Z (2020). HLY78 attenuates neuronal apoptosis via the LRP6/GSK3β/β-catenin signaling pathway after subarachnoid hemorrhage in rats. Neuroscience Bulletin.

[ref-20] Ni M, Xiong M, Zhang X, Cai G, Chen H, Zeng Q, Yu Z (2015). Poly(lactic-co-glycolic acid) nanoparticles conjugated with CD133 aptamers for targeted salinomycin delivery to CD133+ osteosarcoma cancer stem cells. International Journal of Nanomedicine.

[ref-21] Pinczewski J, Obeng RC, Slingluff CL, Engelhard VH (2021). Phospho-β-catenin expression in primary and metastatic melanomas and in tumor-free visceral tissues, and associations with expression of PD-L1 and PD-L2. Pathology Research and Practice.

[ref-22] Ren Z, Li J, Zhao S, Qiao Q, Li R (2021). Knockdown of MCM8 functions as a strategy to inhibit the development and progression of osteosarcoma through regulating CTGF. Cell Death & Disease.

[ref-23] Saini V, Hose CD, Monks A, Nagashima K, Han B, Newton DL, Millione A, Shah J, Hollingshead MG, Hite KM, Burkett MW, Delosh RM, Silvers TE, Scudiero DA, Shoemaker RH (2012). Identification of CBX3 and ABCA5 as putative biomarkers for tumor stem cells in osteosarcoma. PLOS ONE.

[ref-24] Shu X, Liu H, Zhen R, Jie Y, Chen L, Qi H, Wang C, Wang R, Chen D, Ran Y (2020). Hsp90 inhibitor 17‐AAG inhibits stem cell‐like properties and chemoresistance in osteosarcoma cells via the Hedgehog signaling pathway. Oncology Reports.

[ref-25] Singla A, Wang J, Yang R, Geller DS, Loeb DM, Hoang BH (2020). Wnt signaling in osteosarcoma. Yeast Membrane Transport.

[ref-26] Sun S, Fu L, Wang G, Wang J, Xu L (2020). MicroRNA-431-5p inhibits the tumorigenesis of osteosarcoma through targeting PANX3. Cancer Management and Research.

[ref-27] Taciak B, Pruszynska I, Kiraga L, Bialasek M, Krol M (2018). Wnt signaling pathway in development and cancer. Journal of Physiology and Pharmacology.

[ref-28] Tamaddon G, Geramizadeh B, Karimi MH, Mowla SJ, Abroun S (2016). miR-4284 and miR-4484 as putative biomarkers for diffuse large B-Cell lymphoma. Iranian Journal of Medical Sciences.

[ref-29] Tian P, Wang Y, Du W (2021). Ultrasound-targeted microbubble destruction enhances the anti-tumor action of miR-4284 inhibitor in non-small cell lung cancer cells. Experimental and Therapeutic Medicine.

[ref-30] Tornín J, Villasante A, Solé-Martí X, Ginebra MP, Canal C (2021). Osteosarcoma tissue-engineered model challenges oxidative stress therapy revealing promoted cancer stem cell properties. Free Radical Biology and Medicine.

[ref-31] Wang JH, Gong C, Guo FJ, Zhou X, Zhang MS, Qiu H, Chao TF, Liu Y, Qin L, Xiong HH (2020a). Knockdown of STIP1 inhibits the invasion of CD133‐positive cancer stem‐like cells of the osteosarcoma MG63 cell line via the PI3K/Akt and ERK1/2 pathways. International Journal of Molecular Medicine.

[ref-32] Wang T, Wang D, Zhang L, Yang P, Wang J, Liu Q, Yan F, Lin F (2019). The TGFβ-miR-499a-SHKBP1 pathway induces resistance to EGFR inhibitors in osteosarcoma cancer stem cell-like cells. Journal of Experimental & Clinical Cancer Research.

[ref-33] Wang L, Zhou J, Zhang Y, Hu T, Sun Y (2020b). Long non-coding RNA HCG11 aggravates osteosarcoma carcinogenesis via regulating the microRNA-579/MMP13 Axis. International Journal of General Medicine.

[ref-34] Yao J, Lin J, He L, Huang J, Liu Q (2020). TNF-α/miR-155 axis induces the transformation of osteosarcoma cancer stem cells independent of TP53INP1. Gene.

[ref-35] Yin CD, Hou YL, Liu XR, He YS, Wang XP, Li CJ, Tan XH, Liu J (2021). Development of an immune-related prognostic index associated with osteosarcoma. Bioengineered.

[ref-36] Yu T, Liang S, Ma T, Song W (2021). Downregulation of miR-588 is associated with tumor progression and unfavorable prognosis in patients with osteosarcoma. Experimental and Therapeutic Medicine.

[ref-37] Zhang J, Cai H, Sun L, Zhan P, Chen M, Zhang F, Ran Y, Wan J (2018). LGR5, a novel functional glioma stem cell marker, promotes EMT by activating the Wnt/β-catenin pathway and predicts poor survival of glioma patients. Journal of Experimental & Clinical Cancer Research.

[ref-38] Zhang H, Liu S, Tang L, Ge J, Lu X (2021). Long non-coding RNA (LncRNA) MRPL23-AS1 promotes tumor progression and carcinogenesis in osteosarcoma by activating Wnt/β-catenin signaling via inhibiting microRNA miR-30b and upregulating myosin heavy chain 9 (MYH9). Bioengineered.

[ref-39] Zhang Z, Luo G, Yu C, Yu G, Jiang R, Shi X (2019b). MicroRNA-493-5p inhibits proliferation and metastasis of osteosarcoma cells by targeting Kruppel-like factor 5. Journal of Cellular Physiology.

[ref-40] Zhang C, Ma K, Li WY (2019a). IL-6 promotes cancer stemness and oncogenicity in U2OS and MG-63 osteosarcoma cells by upregulating the OPN-STAT3 pathway. Journal of Cancer.

[ref-41] Zhang L, Yang P, Liu Q, Wang J, Yan F, Duan L, Lin F (2020). KLF8 promotes cancer stem cell-like phenotypes in osteosarcoma through miR-429-SOX2 signaling. Neoplasma.

[ref-42] Zhao D, Chen Y, Chen S, Zheng C, Hu J, Luo S (2017). MiR-19a regulates the cell growth and apoptosis of osteosarcoma stem cells by targeting PTEN. Tumour Biology.

[ref-43] Zhao F, Lv J, Gan H, Li Y, Wang R, Zhang H, Wu Q, Chen Y (2015). MiRNA profile of osteosarcoma with CD117 and stro-1 expression: miR-1247 functions as an onco-miRNA by targeting MAP3K9. International Journal of Clinical and Experimental Pathology.

[ref-44] Zhou J, Wu S, Chen Y, Zhao J, Zhang K, Wang J, Chen S (2015). microRNA-143 is associated with the survival of ALDH1+CD133+ osteosarcoma cells and the chemoresistance of osteosarcoma. Experimental Biology and Medicine.

[ref-45] Zhou W, Zhu Y, Chen S, Xu R, Wang K (2016). Fibroblast growth factor receptor 1 promotes MG63 cell proliferation and is associated with increased expression of cyclin-dependent kinase 1 in osteosarcoma. Molecular Medicine Reports.

[ref-46] Zhu M, Wu Y, Wang Z, Lin M, Su B, Li C, Liang F, Chen X (2021). miR-128-3p serves as an oncogenic microRNA in osteosarcoma cells by downregulating ZC3H12D. Oncology Letters.

[ref-47] Zou Y, Huang Y, Yang J, Wu J, Luo C (2017). miR-34a is downregulated in human osteosarcoma stem-like cells and promotes invasion, tumorigenic ability and self-renewal capacity. Molecular Medicine Reports.

