# Supplementary figures and images for: Analysis of microRNA expression in CD133 positive cancer stem‑like cells of human osteosarcoma cell line MG-63
Source: PeerJ. 2021 Sep 3;9:e12115. doi: 10.7717/peerj.12115 (PMC8420872; doi:10.7717/peerj.12115)

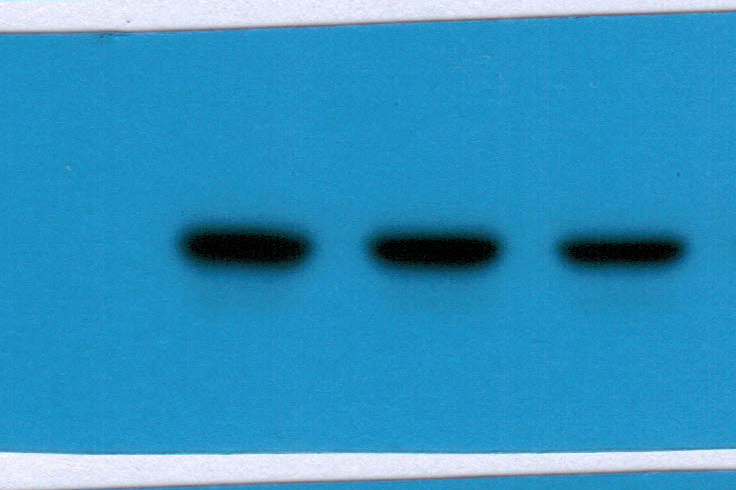

Supplement: Supplemental Information 2 [file peerj-09-12115-s002.zip › Uncropped GelsBlots/beta-cateinin for MG-63.jpg]

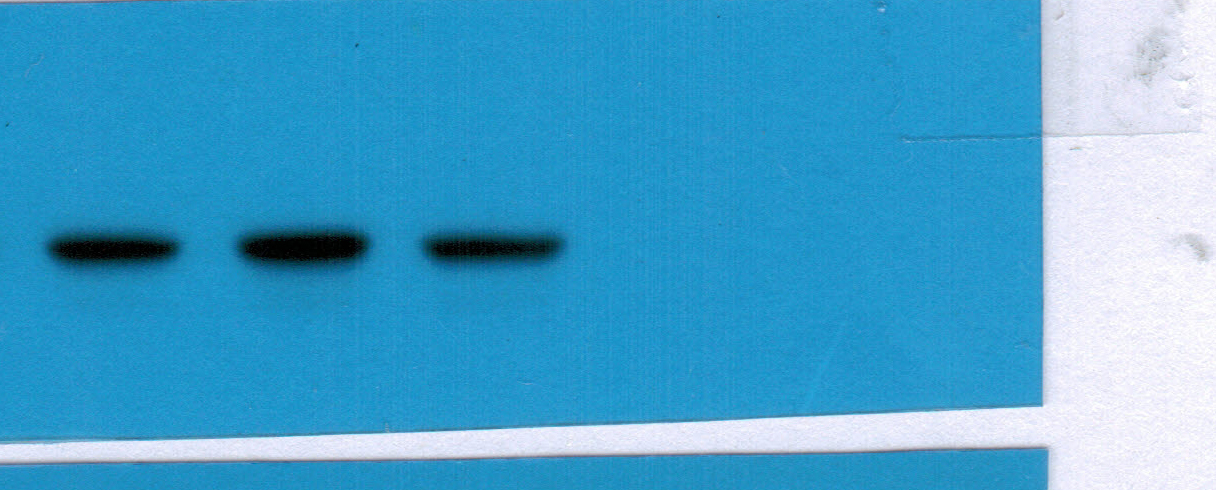

Supplement: Supplemental Information 2 [file peerj-09-12115-s002.zip › Uncropped GelsBlots/beta-cateinin-2 for Soas-2.jpg]

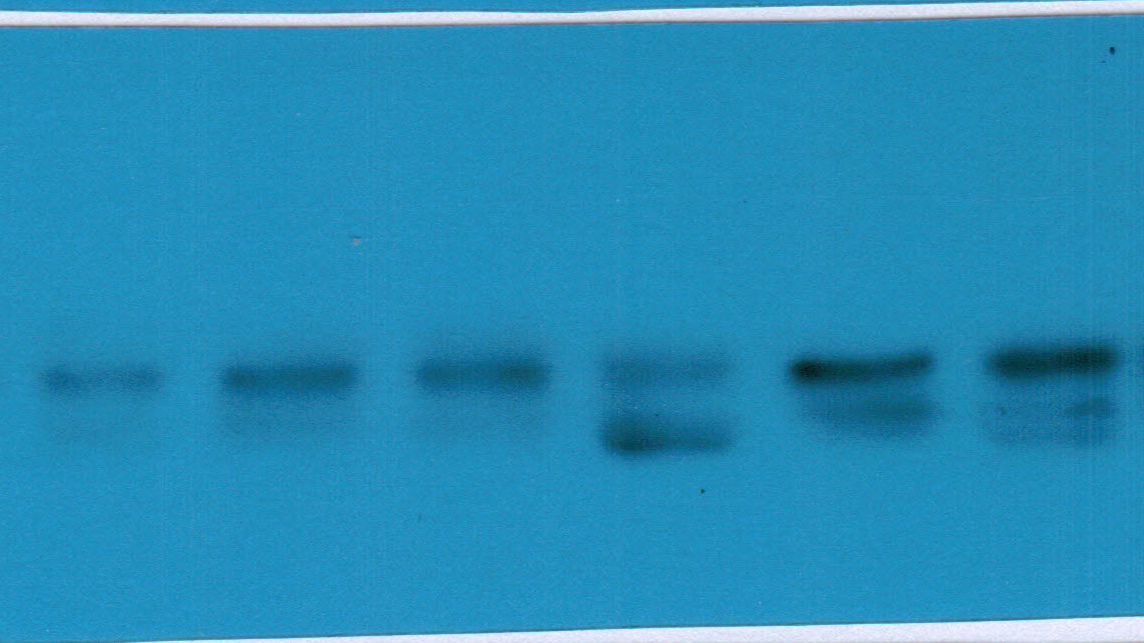

Supplement: Supplemental Information 2 [file peerj-09-12115-s002.zip › Uncropped GelsBlots/p-beta-catenin for MG-63 and Soas-2.jpg]
