# Supplementary material for: Analysis of microRNA expression in CD133 positive cancer stem‑like cells of human osteosarcoma cell line MG-63
Source: PeerJ. 2021 Sep 3;9:e12115. doi: 10.7717/peerj.12115 (PMC8420872; doi:10.7717/peerj.12115)
Supplement: Supplemental Information 3 [file peerj-09-12115-s003.doc]

**Supplementary Table 1. Representative 10 KEGG pathway terms with significantly enriched function for predicted targets of top 3 upregulated miRNAs and top 3 downregulated miRNAs.**

| pathway name | Genes |
| --- | --- |
| Ras signaling pathway | FLT1, CSF1, FLT4, RASGRF2, ETS1, FGF2, IGF1R, FGF5, FGF7, HTR7, TBK1, SYNGAP1, FGF9, AKT3, RAC1, PRKACB, PDGFRA, PLA2G12A, RALBP1, CHUK, PRKCB, PLA2G4E, HGF, RRAS2, PRKCA, KSR2, PGF, TIAM1, MRAS, RASA3, PIK3CA, RASA2, KIT, RAF1, RAPGEF5, MET, SOS2, PLA1A, RALA, RAB5B, SHC3, RAB5C, SHC1, PDGFA, PIK3R1, FOXO4, RASAL2, STK4, PLD1, RASGRP1, EFNA4, EGFR, RELA, RAP1B, CDC42, PAK1, GRIN2A, RAP1A, GNG2, GNG5, PDGFD, PDGFC, FGF20, ABL2, MAPK1, PAK2, PAK5, ANGPT4, PLA2G2D, INSR, PTPN11, IGF1, GNG12, GNG11, GNG13, VEGFA, MAPK10, EFNA3, KITLG, FGF19, GNB1, GNB4, KRAS, CALM3, GNB5, RGL1, CALM1, FGF13, CALM2, RAB5A, LAT, FGFR2 |
| Pathways in cancer | HHIP, ARAF, CBLB, FZD10, ETS1, FGF2, IGF1R, FGF5, FGF7, EDNRB, FGF9, AKT3, SKP2, PRKACB, PDGFRA, RALBP1, TPM3, PRKCB, HGF, WNT5A, FLT3LG, MITF, PRKCA, PGF, RUNX1, AR, COL4A1, MSH3, COL4A6, RAF1, TP53, MAX, CTBP1, TCF7, LPAR1, PDGFA, PIK3R1, STK4, HIF1A, RASGRP1, CSF2RA, FOXO1, DVL2, FGF20, DVL3, FZD1, STAT5B, FZD3, CREBBP, JUN, SMAD4, FZD5, FZD4, PTCH1, FZD7, IGF1, GNG12, GNG11, PTK2, GNG13, NFKBIA, CXCL12, FGF19, GNB1, BCL2, GNB4, GNAS, CYCS, GNB5, FGF13, FGFR2, ITGB1, CDKN1A, CDKN1B, WNT2B, PTEN, SLC2A1, LAMC2, LAMC1, GLI2, MECOM, SUFU, BDKRB2, ITGAV, RAC1, APPL1, HSP90AA1, CHUK, AXIN1, ARNT, FOS, TGFBR1, PLCB4, PIK3CA, KIT, CKS2, RARB, PPARG, PLCB1, MET, SOS2, RALA, FH, LAMA2, ROCK1, ROCK2, HDAC1, GNAI3, XIAP, ADCY1, RELA, ADCY6, EGFR, GNAI1, ADCY5, CDC42, GNG2, RXRA, GNG5, GNA11, MAPK1, E2F3, CTNNA3, VHL, RXRG, RUNX1T1, EGLN1, TCF7L2, TCF7L1, WNT7A, MLH1, VEGFA, MAPK10, KITLG, APC, CCDC6, KRAS |
| PI3K-Akt signaling pathway | CHRM2, CHRM1, CSF1, IRS1, FGF2, IGF1R, FGF5, TCL1B, FGF7, FGF9, AKT3, MYB, CREB3L2, IL6R, IFNAR2, PDGFRA, HGF, TSC1, PRKCA, PRLR, PGF, COL4A1, CDC37, COL4A6, MTCP1, RAF1, TP53, IFNAR1, LPAR1, PDGFA, PIK3R1, FOXO3, EFNA4, BCL2L11, PDGFD, PDGFC, FGF20, MCL1, PCK2, INSR, IGF1, GNG12, GNG11, PTK2, IL2, GNG13, NR4A1, EFNA3, COL5A1, IL7, ITGA10, FGF19, COL5A2, GNB1, BCL2, GNB4, GNB5, FGF13, IL7R, FGFR2, ITGB1, CDKN1A, CDKN1B, FLT1, IFNA6, ITGB5, ITGB4, FLT4, PTEN, LAMC2, LAMC1, BRCA1, GYS2, PPP2R5E, ITGAV, ITGB7, RAC1, JAK2, YWHAH, HSP90AA1, CHUK, PDPK1, ITGA1, PPP2R5D, PPP2R5C, YWHAZ, COL2A1, CREB1, PIK3CA, KIT, ITGA8, COL6A5, SGK1, MET, SOS2, CREB5, PHLPP2, PRKAA1, PRKAA2, LAMA2, THBS2, THBS1, RELA, EGFR, PPP2CA, RELN, GNG2, RXRA, GNG5, MAPK1, EIF4E, EIF4B, ANGPT4, PPP2R3A, VEGFA, KITLG, PPP2R2C, RPS6KB2, IL2RB, KRAS |
| Rap1 signaling pathway | ITGB1, DOCK4, FLT1, CSF1, FLT4, CTNND1, SIPA1L2, SIPA1L3, FGF2, IGF1R, FGF5, FGF7, SIPA1L1, FGF9, AKT3, RAC1, MAGI1, PDGFRA, PRKCB, HGF, MAGI3, MAGI2, PRKCA, PGF, APBB1IP, TIAM1, MRAS, PLCB4, PIK3CA, PRKD3, KIT, RAPGEF2, TLN2, PRKD1, RAF1, RAPGEF5, PLCB1, MET, PFN2, RALA, GNAI3, LPAR1, PDGFA, PIK3R1, ADCY1, THBS1, EFNA4, EGFR, GNAI1, ADCY6, ADCY5, RAP1B, CDC42, PARD6B, GRIN2A, RAP1A, CNR1, PDGFD, PDGFC, FGF20, MAPK1, ANGPT4, INSR, IGF1, MAPK14, MAPK12, VEGFA, GNAO1, EFNA3, KITLG, FGF19, GNAS, KRAS, CALM3, CALM1, FGF13, CALM2, LAT, FGFR2 |
| cGMP-PKG signaling pathway | MYLK2, IRS1, PDE3B, ATP2A2, IRS2, PPP1CB, PPP3CA, PPP1CC, PPP3CB, RGS2, EDNRB, AKT3, CREB3L2, CNGA1, BDKRB2, PRKG1, GTF2I, GUCY1A3, MEF2A, MEF2C, PPP1R12A, ATP1B3, ATP1B2, ATP1B1, ADRA2A, PLCB4, CREB1, KCNMA1, VDAC2, VDAC1, RAF1, PLCB1, SLC25A4, CREB5, ROCK1, ROCK2, SRF, GNAI3, ATP1A2, ATP1A1, ADCY1, GNAI1, ADCY6, ADCY5, PPP3R1, KCNMB1, GNA11, MAPK1, MEF2D, INSR, PDE2A, NFATC3, ATP2B4, NFATC2, NFATC1, ATP2B2, ATP2B1, PDE3A, CALM3, GTF2IRD1, CALM1, CALM2 |
| Wnt signaling pathway | WNT2B, CHD8, FZD10, PPP3CA, PPP3CB, FRAT1, RUVBL1, RAC1, BTRC, PRKACB, PRKCB, FBXW11, WNT5A, AXIN1, PRKCA, DKK1, DKK2, PLCB4, DAAM1, TBL1XR1, PLCB1, TP53, CAMK2B, ROCK2, CTBP1, TCF7, CUL1, PRICKLE2, NLK, CXXC4, LRP6, PPP3R1, DVL2, DVL3, GPC4, CAMK2G, FZD1, TCF7L2, JUN, FZD3, CREBBP, TCF7L1, SMAD4, FZD5, FZD4, CSNK1A1, FZD7, NFATC3, NFATC2, WNT7A, NFATC1, VANGL1, MAPK10, VANGL2, APC, CSNK1A1L |
| Transcriptional misregulation in cancer | CDKN1A, CDKN1B, CCNT2, FLT1, SIX1, JMJD1C, BMI1, AFF1, IGF1R, HHEX, SIX4, SIN3A, ITGB7, WHSC1, SSX2, SSX1, KDM6A, SS18, MEF2C, LMO2, ETV1, PAX5, ETV5, RUNX2, RUNX1, MAF, PPARG, ERG, MET, TP53, DDX5, KMT2A, MAX, HDAC1, PDGFA, MLLT3, FOXO1, RELA, RXRA, TSPAN7, FCGR1A, RXRG, GRIA3, RUNX1T1, SMAD1, CDKN2C, TAF15, H3F3B, EYA1, FUS, PBX3, HMGA2, IGF1, KLF3, PTK2, SSX2B, PER2, MEIS1, NR4A3, BCL6, SP1, ID2, IL2RB, CDK14 |
| Proteoglycans in cancer | ITGB1, CDKN1A, WNT2B, ITGB5, ARAF, CBLB, FZD10, FGF2, IGF1R, PPP1CB, PPP1CC, AKT3, TIMP3, ITGAV, RAC1, PRKACB, PPP1R12A, PDPK1, PRKCB, HGF, WNT5A, RRAS2, ANK2, PRKCA, NUDT16L1, DCN, TIAM1, MRAS, PIK3CA, HCLS1, RAF1, MET, TP53, SOS2, HBEGF, CAMK2B, DDX5, ROCK1, ROCK2, SDC2, PXN, TWIST1, PIK3R1, IQGAP1, THBS1, HIF1A, EGFR, CDC42, PAK1, ERBB4, MAPK1, CAMK2G, EIF4B, FZD1, FZD3, FZD5, FZD4, CAV2, FZD7, PTCH1, RDX, IGF2, WNT7A, PTPN11, IGF1, MAPK14, MAPK12, PTK2, VEGFA, TFAP4, RPS6KB2, PDCD4, KRAS |
| MAPK signaling pathway | RASGRF2, ARRB1, DUSP16, FGF2, FGF5, PPP3CA, RPS6KA3, PPP3CB, FGF7, RPS6KA5, MECOM, FGF9, RPS6KA2, AKT3, RAC1, PRKACB, MAP3K4, MAP2K4, PDGFRA, DUSP2, DAXX, MEF2C, DUSP3, CHUK, PRKCB, PLA2G4E, IL1R1, CACNA2D3, RRAS2, PRKCA, CACNA2D4, FOS, TGFBR1, DUSP6, CACNB2, IL1A, PPM1A, CACNB4, MRAS, RASA2, RAPGEF2, RAF1, TP53, SOS2, MAX, SRF, PDGFA, STK4, NLK, CACNA1E, RASGRP1, EGFR, RELA, STK3, RAP1B, CDC42, CACNG8, PPP3R1, PAK1, RAP1A, NTF3, FGF20, MAPK1, CACNG2, PAK2, MAP4K3, MAP3K2, NTRK2, JUN, MAP3K1, NFATC3, NFATC1, MAPK14, GNG12, MAPK12, MAPK10, NR4A1, TAOK3, TAOK1, FGF19, KRAS, TAB2, FGF13, MAP3K13, PTPN5, LAMTOR3, FGFR2 |
| Axon guidance | ROBO2, SEMA5A, ITGB1, NRP1, LRRC4, RND1, ROBO1, PPP3CA, PPP3CB, DPYSL5, CFL1, RAC1, EPHB1, NCK1, EPHA5, SEMA6C, EPHA7, UNC5A, UNC5C, UNC5D, MET, EPHA3, ROCK1, ROCK2, SEMA3D, NTN4, GNAI3, SEMA3F, EFNA4, GNAI1, EFNB2, CDC42, ABLIM1, PPP3R1, PAK1, MAPK1, SRGAP3, SRGAP2, SRGAP1, PAK2, PAK5, SEMA4B, SEMA4C, NFATC3, NFATC2, L1CAM, PTK2, EFNA3, CXCL12, KRAS, PLXNB1 |
