# Supplementary material for: Analysis of microRNA expression in CD133 positive cancer stem‑like cells of human osteosarcoma cell line MG-63
Source: PeerJ. 2021 Sep 3;9:e12115. doi: 10.7717/peerj.12115 (PMC8420872; doi:10.7717/peerj.12115)
Supplement: Supplemental Information 4 [file peerj-09-12115-s004.doc]

**Supplementary Table 2. Representative 10 KEGG pathway terms with significantly enriched function for validated targets of top 3 upregulated miRNAs and top 3 downregulated miRNAs.**

| **Pathway name** | **Genes** |
| --- | --- |
| Fatty acid metabolism | CPT1A, ACAA2, ACOX1, SCD, SCD5, ACSL6, HSD17B12, HACD4 |
| Morphine addiction | GABRB3, KCNJ6, PDE11A, ADCY9, GNG4, GRK4, PDE4C, GNB4, GNAI3, PRKCA, PDE7A |
| Spliceosome | SF3B3, NCBP2, PRPF4, PRPF6, ZMAT2, SNRPD1, TRA2B, SNRPD3, HNRNPA1, SLU7, SRSF10, SF3B1, RBM22 |
| Biosynthesis of unsaturated fatty acids | ACOX1, SCD, SCD5, HSD17B12, HACD4 |
| Glioma | CDKN1A, CDK4, KRAS, CALM3, PRKCA, IGF1, CALM1, CALM2 |
| Circadian entrainment | KCNJ6, ADCY9, GNG4, GNB4, GNAI3, CALM3, PRKCA, FOS, CALM1, CALM2 |
| Vibrio cholerae infection | ERO1A, ATP6V1A, ATP6V0E1, ADCY9, ATP6V0A2, PRKCA, ACTB |
| Purine metabolism | PNPT1, AK1, PDE4C, GMPS, NTPCR, CECR1, POLA2, PDE11A, ADCY9, NT5C1A, POLR1B, POLR3D, POLR2D, PDE6B, PDE7A |
| p53 signaling pathway | CDKN1A, SESN3, APAF1, CDK4, CYCS, MDM4, IGF1, PPM1D |
| PPAR signaling pathway | CPT1A, PDPK1, ACOX1, SCD, ADIPOQ, SCD5, ACSL6, OLR1 |
